# Use of a Self-Regulation Failure Framework and the NIMH Research Domain Criterion (RDoC) to Understand the Problem of Procrastination

**DOI:** 10.3389/fpsyt.2018.00213

**Published:** 2018-05-29

**Authors:** Idit Shalev

**Affiliations:** Laboratory of Embodiment and Self-Regulation, Department of Psychology, Ariel University, Ariel, Israel

**Keywords:** procrastination, self-regulation failure, RDoC, positive valence systems, cognitive processes, intervention

Although the concept of “doing the right thing at the right time” is fundamental to human adaptive functioning, procrastination, a common and pervasive state of human behavior, may be perceived as a “voluntary delay in an intended course of action despite expecting to be worse off for the delay” [(1), p. 66]. Much of the prior research into procrastination is conceptualized in terms of self-regulation failure, suggesting that individuals experience cognitive and motivational difficulties in goal management (2–5). The term self-regulation refers to control of external behavior and of internal thoughts, emotions, and attention in the process of meeting goals. Accordingly, self-regulation failure results in deficits in cognitive properties of executive function [e.g., (6)], such as task interruption, focus of attention and the contents of working memory (7). The motivational properties of self-regulation failure include failure in action selection, effort valuation and performance, reward learning, reward expectancy and valuation. Finally, a non-conscious element of self-regulation is automatically triggered and often does not require effort (maladaptive habits) (8). Although ample research on procrastination has been conducted in the field of social and personality psychology, little is known about the association between procrastination and the neural circuits that mediate it (9). Researchers have speculated that executive functions play a significant role [e.g., (10)]. Likewise, there is evidence that procrastination is broadly associated with depression and self-criticism (11), which helps explain the possible association between procrastination, self-regulation and specific neural circuits. Based on the National Institute of Mental Health (NIMH) Research Domain Criterion (RDoC), the present paper will examine the association between procrastination and the relevant dimensions of the RDoC. Understanding procrastination as a deficit of the control system together with its link to conditions associated with the same RDoC dimensions may shed light on the neuropsychiatric dimensions aspect of self-regulation failure and help improve the accuracy of procrastination conceptualization and intervention.

## The NIMH research domain criterion (RDoC)

Recent progress in psychiatry suggests that psychopathology refers to a continuum of behavioral functioning—from what is considered the normal adaptive range through degrees of increasing abnormality—and that it should be considered in a neurodevelopmental-environmental context. The vision of the National Institute of Mental Health (NIMH) Research Domain Criterion initiative (RDoC) is to generate research literature about the relations among physiological, behavioral, cognitive, and symptomatic measures that can inform future versions of the International Classification of Diseases (ICD) and the Diagnostic and Statistical Manual of Mental Disorders (DSM) (12). The RDoC initiative proposes to incorporate biological and/or psychometrically advanced measures of behavior and cognition into a precise, diagnostic assessment that will result in treatments tailored to individuals (13). The RDoC purports to examine five potential systems that underlie both normal and abnormal functioning, the latter of which at aberrant levels contributes to the development and maintenance of psychopathology. These domains include negative valence systems, positive valence systems, arousal and regulatory systems, cognitive processes and social processes. The following section will illustrate the possible association between procrastination and the interrelations between the RDoC positive valence system and cognitive processes domains.

## The cognitive systems domain and procrastination

The cognitive systems domain is responsible for a range of cognitive processes, including cognitive control, working memory, declarative memory, attention, perception and language. Much of the literature on procrastination is associated with cognitive control, which modulates the functioning of other cognitive and emotional systems in the service of goal-directed behavior when prepotent modes of responding are inadequate to meet the demands of the current context. For example, there is evidence that executive functions, a set of goal-related cognitive abilities, are linked with procrastination. General control mechanisms implicated in goal-related abilities, executive functions are considered to constitute a critical component of self-control (14, 15). The scientific literature suggests that a common executive function be defined, namely, one that relies on the ability to activate and maintain appropriate goals and, in so doing, it effectively guides lower level processes. This approach can help explain various findings indicating an association between procrastination and conscious executive functions (9). For example, there is evidence that procrastination and impulsivity share identical genetic influences (i.e., a genetic correlation of 1.02) and that goal management abilities also account for most of this shared variation at the genetic level (about 68%). These results extend the goal-management accounts of procrastination by suggesting that these traits are connected due to their shared genetic influences (3).

## Implications for therapy

Several interventions could be associated with the cognitive processes domain of the RDoC, which indicates the importance of conscientiousness (16). Among these interventions are time management (TM) strategies, such as setting deadlines [e.g., (17)], monitoring and reporting compliance with deadlines (18), creating specific plans for goal completion [e.g., (19, 20)], and learning study skills (21) for task completion. This approach, however, was not effective for all participants. For example, the findings indicate that the study skills intervention was the least effective strategy for students with a high tendency to procrastinate (21). Furthermore, across studies, TM strategies were shown to be not effective for all tasks. For example, setting implementation intentions appears to be more effective at helping participants complete “difficult” than “easy” goals (19). Based on these findings, interventions using acceptance and mindfulness methods to increase psychological flexibility were compared time management and acceptance-based behavioral interventions. College students' predictions of how much of their assigned reading they should complete were compared with what they actually completed. Although the results showed a trend suggesting that participants who practiced time management intervention completed more reading, and no group differences in procrastination were revealed (22). These findings demonstrate the heterogeneous nature of procrastination, indicating the possible contribution that positive valence systems could make to our understanding of procrastination.

## Positive valence systems and procrastination

The RDoC's notion of positive valence systems is associated with the motivational dimension of self-regulation and includes several components. The first is approach motivation, a multi-faceted construct involving mechanisms that regulate the direction and maintenance of approach behavior including pre-existing tendencies, learning, memory and environmental cues that can be directed toward goals (23). Research has demonstrated that an individual's goal-setting ability underlies the tendency to procrastinate (24). Similarly, there is evidence that deficits in promotion goals impair adaptive functioning in multiple arenas (25–27). Individuals who were unable to pursue promotion goals effectively were at risk for mood disorders via a common pathway (26–28). Research following this view indicates an association between low promotion goals and depression (27); in contrast, there is evidence of a relation between locomotion tendency and well-being (29). A second set of components included in positive valence systems comprises the dynamics of rewards. For example, initial reward responsiveness includes mechanisms associated with hedonic responses, demonstrating that procrastination is associated with task aversiveness, boredom, uncertainty, and guilt [(30–32)]. Likewise, sustained or longer-term responsiveness to reward attainment (e.g., termination of reward seeking, satisfaction or satiation) suggests that the cause for procrastination is the primacy of short-term reinforcement and mood repair over long-term goal pursuit (4, 33). Reward learning behavior is related to the association that organisms form between stimuli, behavior and reward, a notion that helps explain the relation between procrastination, learned helplessness and depression. This also helps explain recent findings indicating that dopamine-receptor signaling while conductingan aversive contextual assignmentregulates aversive memory retention and regulates associated synaptic mechanisms in the hippocampus that likely underlie learning (34). Finally, a third and important component of positive valence systems, habit, refers to sequential and repetitive motor and/or cognitive behaviors elicited by external or internal triggers that, once initiated, can continue to completion in the absence of constant, conscious oversight. There is evidence of a negative relation between intention and procrastination, such that people who recurrently procrastinate do not necessarily lack an intention to initiate or complete their tasks or assignments, but rather, they act automatically, and acting on their intentions is a difficult challenge for them (35).

## Implications for therapy

Use of the RDoC's positive valence systems concept enables the possible association between procrastination, learned helplessness and depression to be reexamined. Although ample research has demonstrated the efficacy of behavioral activation intervention in the treatment of depression, little is known about whether the same principles could be used to target procrastination. Behavioral activation and effort-related processes are fundamental components of the construct of motivation. Behavioral activation intervention is based on the notion that a deficit in the behavioral activation system is related to the number of potentially reinforcing events one experiences, the availability of reinforcement in the environment, and the ability to obtain the available reinforcement (36). Therefore, behavioral activation intervention stimulates a process in which increasing the frequency of one's overt behaviors facilitates exposure to reinforcing environmental contingencies, which, in turn, leads to subsequent reductions in behavioral dysfunction (37–39). To facilitate action initiation, an integral part of behavioral activation intervention comprises the identification of personal values, the creation of a hierarchy of the association between values and activity, and activity scheduling and monitoring (39).

This paper is the first to conceptualize procrastination as a self-regulation failure within the scope of the NIMH RDoC domains. The varied patterns of procrastination that have been observed could be associated with correspondingly different patterns of self-regulation failure, indicating that procrastination is a heterogeneous trait (40). It appears that control system dysfunction may be a common factor cutting across a broad range of conditions [(29, 41)]. Based on the reasoning that psychopathology describes a continuum of behavioral functioning—from the normal, adaptive range through degrees of increasing abnormality—procrastination may be associated with other conditions with different levels of severity (e.g., depression) that may share similar treatment protocols (e.g., behavioral activation). Because some patterns of self-regulation failure are related to similar brain circuits, the treatment of procrastination may be related to the treatment of other conditions associated with the similar brain circuits. The cognitive properties of executive function in procrastination (e.g., 6) as well as implicit and explicit motivational properties (42) comprise two aspects of self-regulation failure that are influenced by neurobiological pathways, behavioral patterns and the social environment, thus illustrating the importance of social and personality psychology in enriching our understanding of mental states and conditions.

## Author contributions

The author confirms being the sole contributor of this work and approved it for publication.

### Conflict of interest statement

The author declares that the research was conducted in the absence of any commercial or financial relationships that could be construed as a potential conflict of interest.
